# A method to assess image quality for Low-dose PET: analysis of SNR, CNR, bias and image noise

**DOI:** 10.1186/s40644-016-0086-0

**Published:** 2016-08-26

**Authors:** Jianhua Yan, Josh Schaefferkoette, Maurizio Conti, David Townsend

**Affiliations:** 1 Department of Nuclear Medicine, First Hospital of Shanxi Medical University, 85 Jiefang S Rd, Yingze, Taiyuan, Shanxi 030001 China; 2 Molecular Imaging Precision Medicine Collaborative Innovation Center, Shanxi Medical University, 85 Jiefang S Rd, Yingze, Taiyuan, Shanxi 030001 China; 3 A*STAR-NUS, Clinical Imaging Research Center, Center for translational medicine, 14 medical drive, #B1-01, 17599 Singapore, Singapore; 4 Department of Diagnostic Radiology, National University Hospital, Main Building, 5 Lower Kent Ridge Road, Level 3, 119074 Singapore, Singapore; 5 Siemens Healthcare Molecular Imaging, 810 Innovation Drive, Knoxville, TN37932 USA

**Keywords:** Low dose, PET/MR, PET/CT, Lung, Image quality

## Abstract

**Background:**

Lowering injected dose will have an effect on PET image quality. In this article, we aim to investigate this effect in terms of signal-to-noise ratio (SNR) in the liver, contrast-to-noise ratio (CNR) in the lesion, bias and ensemble image noise.

**Methods:**

We present here our method and preliminary results using tuberculosis (TB) cases. Sixteen patients who underwent ^18^F-FDG PET/MR scans covering the whole lung and portion of the liver were selected for the study. Reduced doses were simulated by randomly discarding events in the PET list mode data stream, and ten realizations at each simulated dose were generated and reconstructed. The volumes of interest (VOI) were delineated on the image reconstructed from the original full statistics data for each patient. Four thresholds (20, 40, 60 and 80 % of SUVmax) were used to quantify the effect of the threshold on CNR at the different count level. Image metrics were calculated for each VOI. This experiment allowed us to quantify the loss of SNR and CNR as a function of the counts in the scan, in turn related to dose injected. Reproducibility of mean and maximum standardized uptake value (SUVmean and SUVmax) measurement in the lesions was studied as standard deviation across 10 realizations.

**Results:**

At 5 × 10^6^ counts in the scan, the average SNR in the liver in the observed samples is about 3, and the CNR is reduced to 60 % of the full statistics value. The CNR in the lesion and SNR in the liver decreased with reducing count data. The variation of CNR across the four thresholds does not significantly change until the count level of 5 × 10^6^. After correcting the factor related to subject’s weight, the square of the SNR in the liver was found to have a very good linear relationship with detected counts. Some quantitative bias appears with count reduction. At the count level of 5 × 10^6^, bias and noise in terms of SUVmean and SUVmax are up to 10 and 20 %, respectively. To keep both bias and noise less than 10 %, 5 × 10^6^ counts and 20 × 10^6^ counts were required for SUVmean and SUVmax, respectively.

**Conclusions:**

Initial results with the given data of 16 patients diagnosed as TB demonstrated that 5 × 10^6^ counts in the scan could be sufficient to yield good images in terms of SNR, CNR, bias and noise. In the future, more work needs to be done to validate the proposed method with a larger population and lung cancer patient data.

## Background

PET is an important tool for in-vivo study of quantitative measurements of physiological, biochemical, or pharmacological processes. In thoracic oncology, ^18^F-FDG PET currently plays a major role in clinical diagnosis, staging, prognosis and assessment of response to treatment [1]. Recent advances in PET and CT technology have improved image quality while reducing radiation exposure to patients [2, 3], which open new avenues for cancer screening with PET and CT. Several studies showed that low dose CT is superior to traditional chest radiography for lung cancer screening and follow-up by detecting more nodules and lung cancers including early-stage cancers [4, 5]. In addition, low dose CT screening for subjects at high risk could reduce lung cancer mortality [6]. However, due to its limited specificity, low dose CT screening also detected more than 18 % of all lung cancers which were indolent and led to overdiagnosis in screening for lung cancer [7] although computer-aided diagnosis could improve performance of CT screening [8]. A large clinical study on CT screening of patients at risk has shown that even if cancer mortality is reduced by low dose CT screening, however, 24.2 % of the patients were tested positive, but 96.4 % of these were false positives [6]. This large number of false positives calls for imaging techniques with higher specificity, in order to avoid unneeded invasive biopsy. Additional metabolic information from ^18^F-FDG PET has been shown to be more specific than CT in detecting lung cancer [9]. Moreover, the combination of CT and PET demonstrated better performance in classifying solitary pulmonary nodules as benign or malignant than either PET or CT alone [8]. Thus, the synergetic effect of PET and CT could potentially improve the accuracy of screening for lung cancer [10]. Like low dose CT screening, the radiation exposure due to injected isotope should be minimized without compromising image quality of PET. The effective dose associated with ^18^F-FDG PET exam in this study was computed based on the reported ICRP values of 0.019 mSv/MBq for a 70 kg adult. For example, the effective dose is about 7 mSv for typical administration of 10 mCi ^18^F-FDG, which is much higher than that (1.5 mSv) of low dose CT protocol used in the National Lung Screening Trial [11]. The continual improvement of PET imaging, such as introduction of point spread function and time of flight technologies, could allow for lower injected activities while minimizing impact on image quality [12].

A number of studies to investigate the effect of different count levels on PET image quality with phantom have been reported [13, 14]. Our previous study demonstrated count statistics as low as 5 × 10^6^ counts could achieve a fairly high detectability level using a data set of ^18^F-FDG PET images of tuberculosis (TB) patients acquired on a PET/MR scanner [15]. In this work, we aimed to assess the relationship between numbers of counts in PET scan and image quality with these data, based on image metrics such as liver signal-to-noise ratio (SNR), lesion contrast-to-noise ratio (CNR), bias relative to the “true value”, and ensemble noise in the image (lesion and normal tissue).

## Methods

### Data acquisitions

Sixteen patients with TB (male: 12, female: 4) having a mean age of 45 years (range: [24–67]), a mean weight of 58.53 kg (range: [45–79]) and a mean BMI of 19.78 (range: [15.21–26.70]) underwent ^18^F-FDG PET/MR scans at A*STAR-NUS, Clinical Imaging Research Center using a Siemens mMR PET/MR scanner. The scanning usually started after the FDG uptake time of 60 min but were subject to the availability of the scanners. In addition, four patients had one PET/CT scan and one PET/MR scan but with one ^18^F-FDG injection. The interval between the two scans was around 60 min and the order of these two PET scans was random. However, only the PET/MR scan was included in this study. The PET spatial resolution of mMR has been measured, at 1 and 10 cm from the center of the field of view (FOV), as 4.3 mm and 5.2 mm transaxially, and 4.3 mm and 6.6 axially [16]. The coincidence timing window is 5.9 ns and the energy window is 435–650 keV. The detector system includes 8 rings of 56 detector blocks, each comprising an 8 × 8 matrix of lutetium oxyorthosilicate crystals (4 × 4 × 20 mm), coupled to an array of 3 × 3 APDs. The axial PET FOV is 25.2 cm, and all emission data were acquired in 3D mode. The data were organized into separate prompts and delayed sinograms. The delayed events were smoothed and used to estimate the overall random coincidences. All patients fasted at least for 8 h with serum glucose level less than 10 mmol/L. The time difference between injection and acquisition was 80.1 ± 26.2 min (range: [36.5–126.4] min) after injection of 168.6 ± 50.0 MBq (range: [118.0–260.5] MBq) ^18^F-FDG, with 15 min for one bed position covering the whole lung and portion of the liver. The true coincident events, after random subtraction, were 1.32 × 10^8^ ± 3.91 × 10^7^ (range: [8.33 × 10^7^–2.00 × 10^8^]).

### Data reconstructions

The reduced doses were simulated by randomly discarding events in each list mode stream according to 10 predefined fractions of original net true counts: 5 × 10^−1^, 2.5 × 10^−1^, 1.25 × 10^−1^, 6.25 × 10^−2^, 3.33 × 10^−2^, 1.67 × 10^−2^, 5 × 10^−3^, 3.33 × 10^−3^, 1.67 × 10^−3^, and 5 × 10^−4^. The random events in the list mode data stream were discarded using the same procedure as resampling true events. Ten independent realizations at each simulated dose were generated. Each realization was reconstructed with ordinary Poisson ordered subsets expectation maximization (OP-OSEM) with a system point spread function (PSF) incorporated in the projection matrix [17]. Corrections including attenuation, randoms, and scatter were carried out for each realization. Three iterations and 21 subsets were used to produce image matrices of 172 × 172 × 127 with voxel sizes of 4.17 × 4.17 × 2.03 mm. The image volumes were then smoothed with a 5 mm Gaussian filter.

### VOI delineation

In this study, the mean standardized uptake value (SUVmean) was calculated as ^18^F-FDG uptake normalized to injected dose and patient body weight and activity was decay corrected. Volume of interest (VOI) around solitary lung lesions were delineated using fixed threshold set to 40 % of the maximum standardized uptake value (SUVmax) in the lesion and followed by a manual adjustment to exclude neighboring nodes for each VOI if there was any in the VOI. This thresholding was used to investigate the effect of count level on CNR in the lesion, lesion bias with reference to SUV calculated in the images at the full statistical count level, noise across realizations and reproducibility of the SUVmean and SUVmax in the lesion. Twenty small lesions were obtained from these 16 subjects and the volume was 6.38 ± 4.75 ml (range: [1.2−17.58 ml]). The VOIs in the normal lung background and liver background were obtained by drawing spheres with diameter of 3 cm in these two regions. All of the VOIs were delineated on the images reconstructed with the original full statistics data.

### Image analysis

For the evaluation of image quality, five metrics were used 1) SNR in the liver, 2) CNR in the lesion, 3) bias with reference to SUV calculated in the images at the full statistics count level, 4) noise in the image expressed as percentage coefficient of variation (COV) in VOIs including lesion, normal lung and liver, across 10 realizations, and 5) error or reproducibility of the SUVmean and SUVmax in the VOIs including lesion, normal lung and liver, across 10 realizations.

1. The signal to noise ratio (SNR) was calculated as the ratio of mean value to standard deviation (SD) in the VOI:
  1 $$ Mean(VOI)=\frac{{\displaystyle \sum_{j\in VOI}{I}_{mean}(j)}}{N_{VOI}} $$
  2 $$ SD(VOI)=\sqrt{\frac{{\displaystyle \sum_{j\in VOI}{\left({I}_{mean}(j)- Mean(VOI)\right)}^2}}{N_{VOI}}} $$
  3 $$ SNR(VOI)=\frac{Mean(VOI)}{SD(VOI)} $$
  where *Mean*(*VOI*) and *SD*(*VOI*) are mean value and SD value of the *VOI* in the mean image (*I*_*mean*_) across the realizations, respectively, *N*_*VOI*_ is the number of voxels in the *VOI. VOI* could be in the lesion, clear lung background and liver. The SNR in the liver was widely used to quantify ^18^F-FDG PET image quality due to its relatively homogeneous uptake. Due to the Poisson statistics of nuclear positron emission, the SNR of PET images depends on the injected activity, the acquisition time, and the attenuation [18, 19], and it can be expressed as follows:
  4 $$ SN{R}_{liver}^2\approx N\approx {K}_i\bullet D\bullet t $$
  5 $$ {K}_i=k\bullet \left(g\bullet \eta \bullet \frac{1}{a(m)}\right) $$
  where *N* is the number of detected counts, *K*_*i*_ is a composite sensitivity factor, *D* is the injected activity and *t* is the scan time, *k* is a proportionality constant, *g* is a noise reduction factor due to reconstruction techniques such as PSF and time-of-flight, *η* is the scanner sensitivity, and *a*(*m*) is the attenuation factor, a function of subject’s weight *m*. The corrected SNR^2^ in the liver can be made independent of patient dividing by *K*_*i*_.
2. The contrast to noise ratio (CNR) is a measure of the signal level in the presence of noise given by [20]:
  6 $$ CNR=\frac{Mean(lesion)- Mean(background)}{SD(background)} $$
  where background is measured in the neighboring normal lung tissue, *Mean*(*lesion*) and *Mean*(*background*) are the mean value of the lesion and background region in the mean image of the SUV across the realizations calculated with Eq. 1, respectively and *SD*(*background*) is the SD value of the background region in the mean image across the realizations calculated with Eq. 2. The background mask was obtained by performing morphological operation on the lesion mask:
  7 $$ backgroundmask= dilation\left( lesionmask,2\right)- lesionmask $$
  where *dilation*(*lesionmask*, 2) means dilating the lesion mask by 2 voxels.
3. Bias describes the difference of estimated SUV from the true value, which is unknown in the clinic. In this work, the percentage difference relative to the activity uptake at full statistics count level is used to estimate bias.
  8 $$ Bia{s}_{frac}\left(\%\right)=\frac{\left(Mea{n}_{frac}(VOI)-Mea{n}_{full}(VOI)\right)}{Mea{n}_{full}(VOI)}*100 $$
  where *Bias*_*frac*_ represents the bias in percent at the predefined fraction of counts, *Mean*_*frac*_ is the mean value of the *VOI* in the mean image (*I*_*mean*_^*frac*^) across the realizations at the predefined fraction count level. The *Mean*_*full*_(*VOI*) is the mean value of the *VOI* in the image at the full count level (*I*_*mean*_^*full*^), typically greater than 100 × 10^6^ counts. Both *Mean*_*frac*_ and *Mean*_*full*_(*VOI*) were calculated with Eq.1.
4. The coefficient of variation (COV) is a metric for describing ensemble noise or statistical noise in the image, and it can influence the detectability of the lesion.
  9 $$ CO{V}_{frac}(VOI)=\frac{Mea{n}_{mean}^{frac}(VOI)}{Mea{n}_{SD}^{frac}(VOI)}*100 $$
  where *Mean*_*mean*_^*frac*^(*VOI*) and *Mean*_*SD*_^*frac*^(*VOI*) are the mean value of the *VOI* in the mean image and the SD image across the realizations at the predefined fraction count level calculated with Eq.1, respectively.
5. Standard error (STE) is a metric for describing error on the measurement of SUV, and it can represent the reproducibility of the measurement.
  10 $$ ST{E}_{frac}\left(\%\right)=\frac{S{D}_{frac}\left(Mea{n}_{frac}(VOI)\right)}{Mea{n}_{frac}(VOI)}*100 $$
  where *SD*_*frac*_ is the standard deviation (across the realizations) of the SUV in the *VOI* at the predefined fraction count level, normalized to the SUV averaged across realizations.
6. The effect of different thresholding (20, 40, 60 and 80 % of SUVmax) on CNR at different count level was investigated. In this comparison, the surrounding background mask delineated with 20 % of SUVmax thresholding was applied to the other three thresholding. Each CNR was calculated from the mean image across realizations with Eq. 6. COV of each CNR was calculated across the four thresholding to demonstrate the sensitivity of the CNR to the thresholding.
  The bias, COV, and STE were studied as a function of the counts in the scan, in the lung lesions, in the lung background, and in the liver, in the pooled sample of patients and lesions. For each count level we histogrammed the number of data point that pass a given threshold (in percent), which refers to either bias, COV, or STE for SUVmean and SUVmax. We used the mean of the histogram distribution to define the number of counts associated with such percent average bias (or COV, or STE). We finally plotted counts vs. percent variation for SUVmean or SUVmax bias (or COV, or STE) for TB lesions, background lungs and uniform liver region. This would allow a quantitative assessment of the number of counts needed for a given acceptable error in the measurement, systematic or statistical.

## Results

### Effect of counts on SNR in the liver

**Fig. 1:**
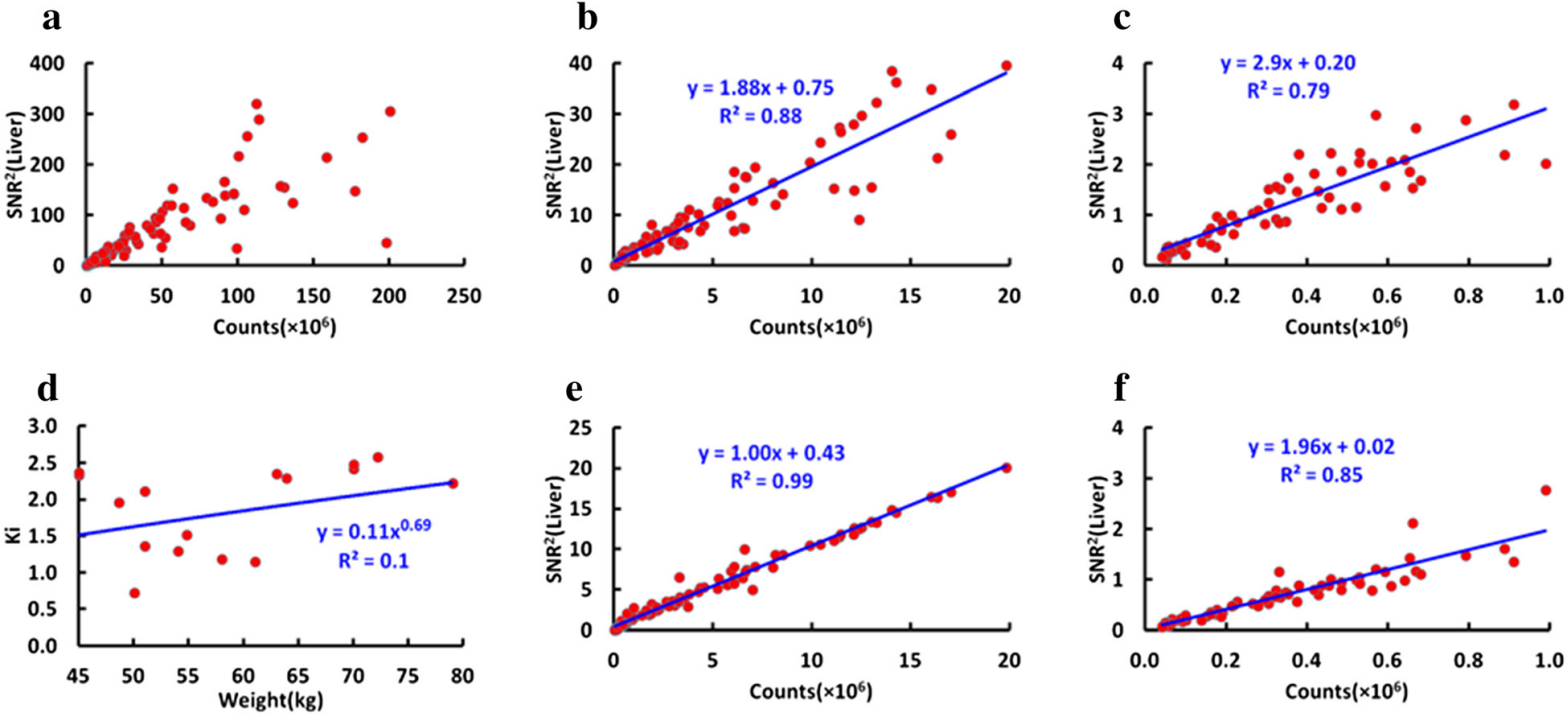
**a** The SNR^2^ in the liver for all the reconstructed images; (**b**) The SNR^2^ in the liver for the images reconstructed with fewer than 20 × 10^6^ true counts; (**c**) The SNR^2^ in the liver for the images reconstructed with fewer than 1 million true counts; (**d**) The composite sensitivity factor *Ki* for all subjects and the corresponding fitted curve; (**e**) The corrected SNR^2^ in the liver for the images reconstructed with fewer than 20 million true counts and the linearly fitted curve; (**f**) The corrected SNR^2^ in the liver for the images reconstructed with raw data less than 1 × 10^6^ true counts and the linearly fitted curve

The average SUV_mean_ in the liver for the images reconstructed with the original full statistics data was 1.57 ± 0.40 (range: [0.54–2.12]). The SNR^2^ in the liver for the images with full statistics, for the images with fewer than 20 × 10^6^ true counts, and for the images with fewer than 1 × 10^6^true counts are shown in Fig. 1(a, b and c), respectively. The *K*_*i*_ for each subject was obtained by fitting the SNR^2^ with the number of counts (*y* = 0.11*x*^0.69^, *R*^2^ = 0.1), according to Eq. (4), and is shown in Fig. 1(d). The SNR^2^ corrected by the *K*_*i*_ are shown in Fig. 1(e and f) with true counts less than 20 × 10^6^ and 1 × 10^6^, respectively. One can see that the corrected SNR^2^ has better linear relationship with detected counts than the original SNR^2^.

In PET imaging, photons registered in the detectors follow a Poisson distribution and the standard deviation on the number of counts *N* is proportional to the square root of *N*, therefore, SNR^2^ of the counts registered in the detectors is linear with *N.* Iterative reconstruction method based on maximum likelihood estimate is the most popular method in the most of PET scanner, which is a nonlinear method and leads to the non-linear relationship of the SNR^2^ measured with the reconstructed image with *N*. However, in the case of high statistics, the nonlinear transformation can be approximate to a linear one. As expected, the proportionality coefficient in the mid-range of 1–20 × 10^6^ counts is 1 (Fig. 1(e)), but at extremely low counts such proportionality does not hold anymore, and applying a linear fit the coefficient is now 1.96 (Fig. 1(f)). This represents a loss of proportionality at low counts, when Poisson statistics does not represent the data well anymore, and the relationship between counts and SNR^2^ is not linear.

### Effect of counts on CNR in the lesion

**Fig. 2:**
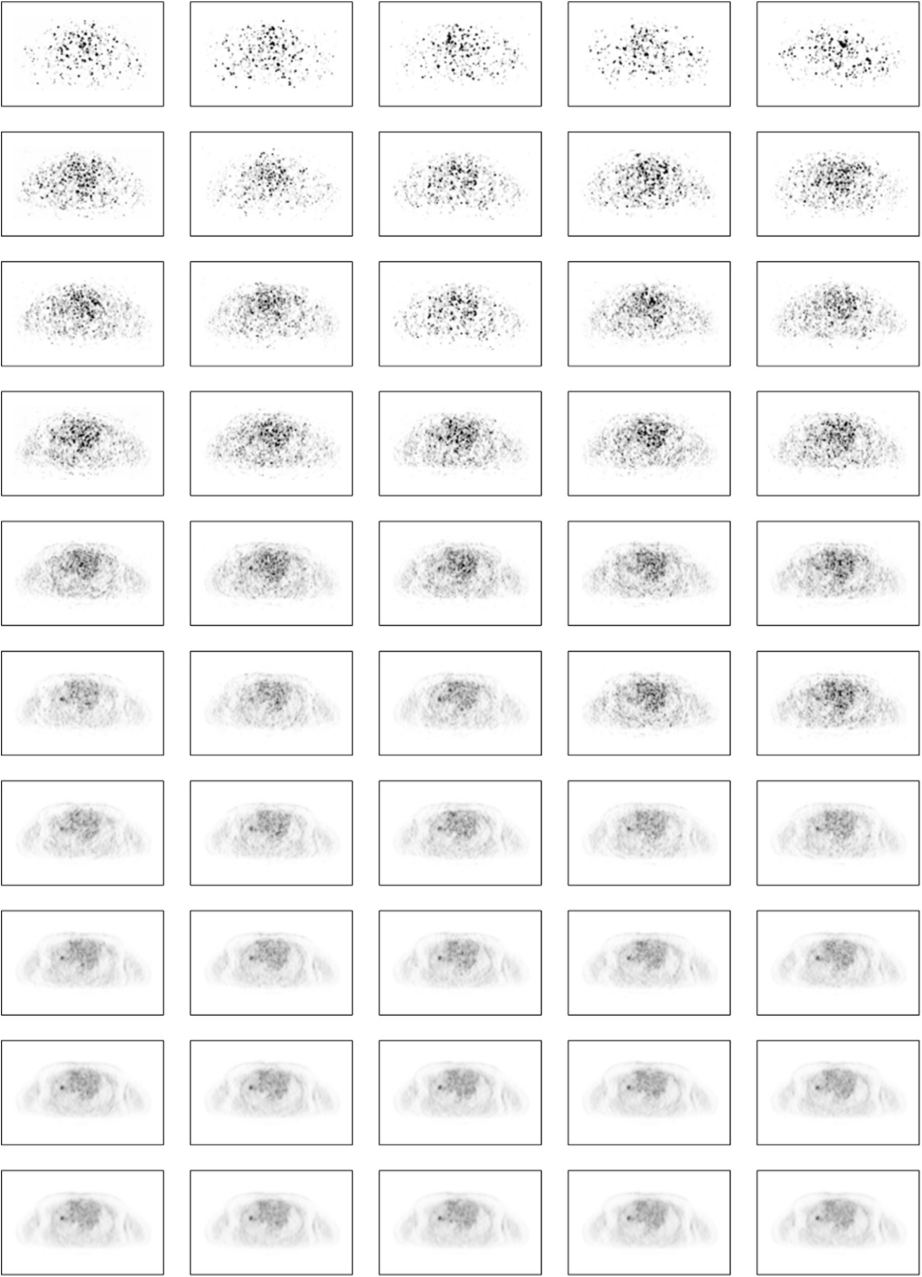
Typical PET images (SUV: 0–6) with a lesion of CNR (=3.17) and volume (1.66 ml) at the different count level (5 realizations are displayed). Each column represents one realization. The count level corresponding to each row is 0.048 × 10^6^, 0.16 × 10^6^, 0.32 × 10^6^, 0.48 × 10^6^, 1.6 × 10^6^, 3.2 × 10^6^, 6 × 10^6^, 1.2 × 10^7^, 2.4 × 10^7^, 4.8 × 10^7^

Twenty six lesions were delineated by 40 % of SUV_max_ thresholding method and the lesions with volume less than 20 ml, corresponding to approximate 3.37 cm of diameter for spherical lesion, were found in these 16 subjects with average SUV_mean_ of 1.92 ± 0.95 (range: [0.92–4.81]). Typical PET images with five realizations with a lesion of CNR of 3.17 and volume of 1.66 ml at the different count level are shown in Fig. 2. As expected, image quality decreases with decreasing counts. The lesion can be visually detected in the image at the count level of more than 3 × 10^6^. Since it is challenging to reliably identify lesion with low CNR, especially at low count level, lesions with CNR less than 2 were excluded from this study. Twenty lesions were found to satisfy the requirement of volume (≤20 ml) and CNR (≥2).

**Fig. 3:**
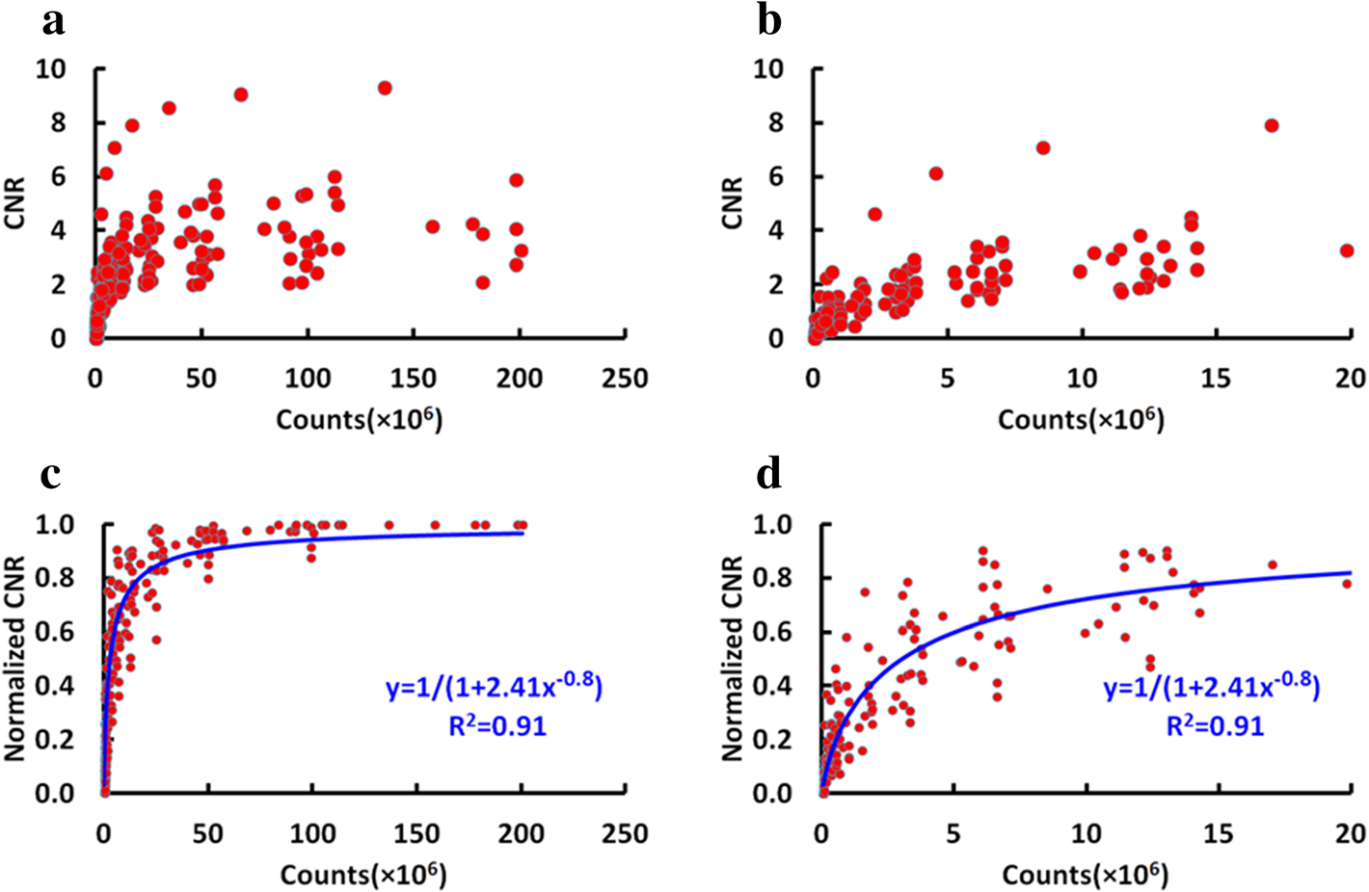
Plot of CNR at different count levels. **a** CNR at the full count range, (**b**) CNR at counts fewer than 20 × 10^6^, (**c**) normalized CNR (CNR normalized by the value at full count level) at the full count range and the fitted function, (**d**) normalized CNR at counts fewer than 20 × 10^6^ and the fitted function

Figure 3(a) shows CNR of all lesions at different count levels and Fig. 3(b) displays CNR at the count level range of 0–20 × 10^6^. CNR decreases with the lowering of count. All CNR were normalized to the value at the full count level and are shown in Fig. 3(c and d). Normalized CNR can be fitted with count by a function (y = 1/(1 + 2.41x^− 0.8^), R^2^ = 0.91). This curve allows to predict the amount of counts required to obtain a desired normalized CNR.

### Effect of thresholding on CNR

**Fig. 4:**
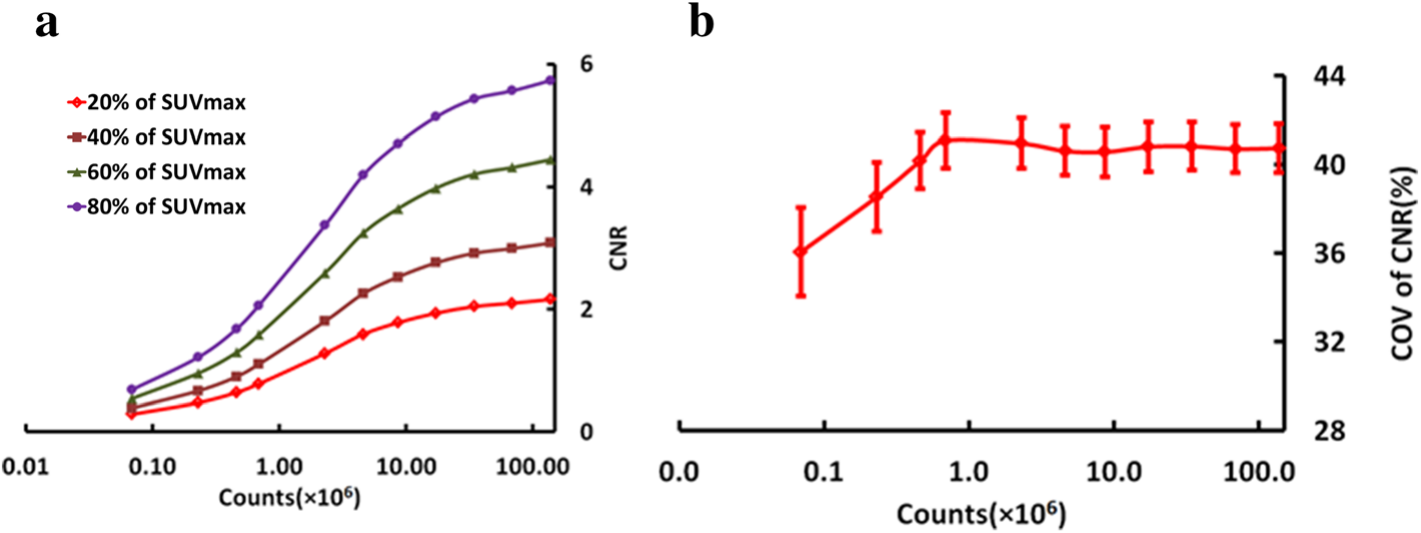
Effect of different threshold on CNR at the different count level: (**a**) the mean CNR of all lesions at different count level. **b** the mean COV of all lesions’ CNR across the realizations. The variation over the realizations is demonstrated as the error bar

Figure 4(a) shows the mean CNR of all lesions at different count level using different thresholding method. It can be seen that higher thresholding method produces higher CNR. In addition, all lesions’ CNR decrease with lowering counts. Figure 4(b) demonstrates the mean COV of all lesions’ CNR across the realizations, which indicates that the mean COV do not significantly vary until a count level of 5 × 10^6^.

### Effect of counts on bias and COV

**Fig. 5:**
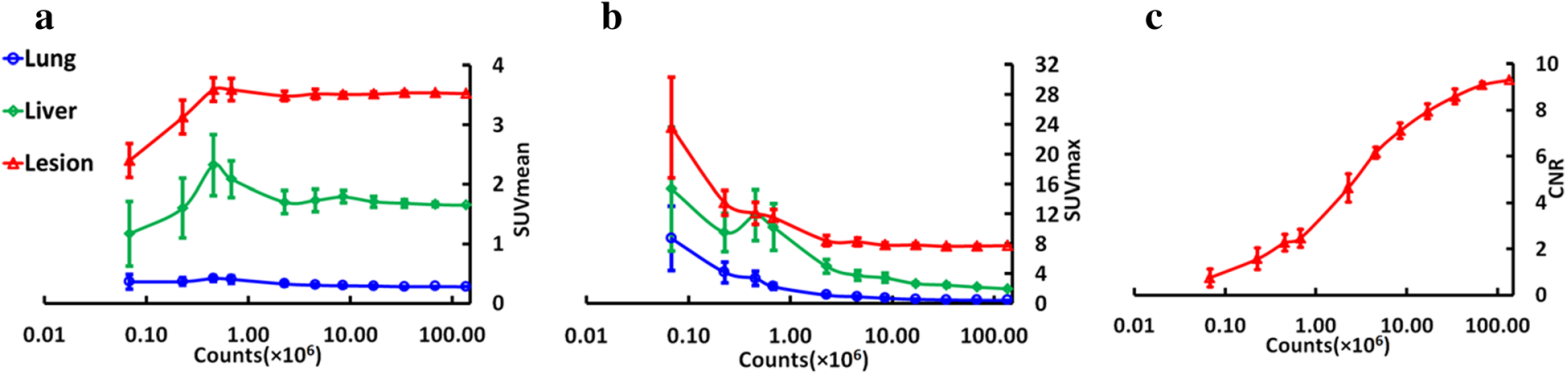
Example of SUV and CNR vs. counts (patient as in Fig. 4): (**a**) SUV_mean_ in the normal lung (blue), liver (green) and TB lesion (red) at the different count level. **b** SUV_max_ in the normal lung (blue), liver (green) and TB lesion (red) at the different count level. **c** CNR in the lesion at the different count level. The variation over the realizations is demonstrated as the error bar

Figure 5(a and b) shows the mean SUV_mean_ and SUVmax in the normal lung, liver and lesion, respectively. Figure 5(c) exhibits the CNR in the same lesion at variable count levels for the same patient. One can see that the SUV_mean_ in these three regions for this subject do not significantly change, relative to the full count level, down to a count level of 1 × 10^6^. For the SUVmax, the count level is around 8 × 10^6^.

**Fig. 6:**
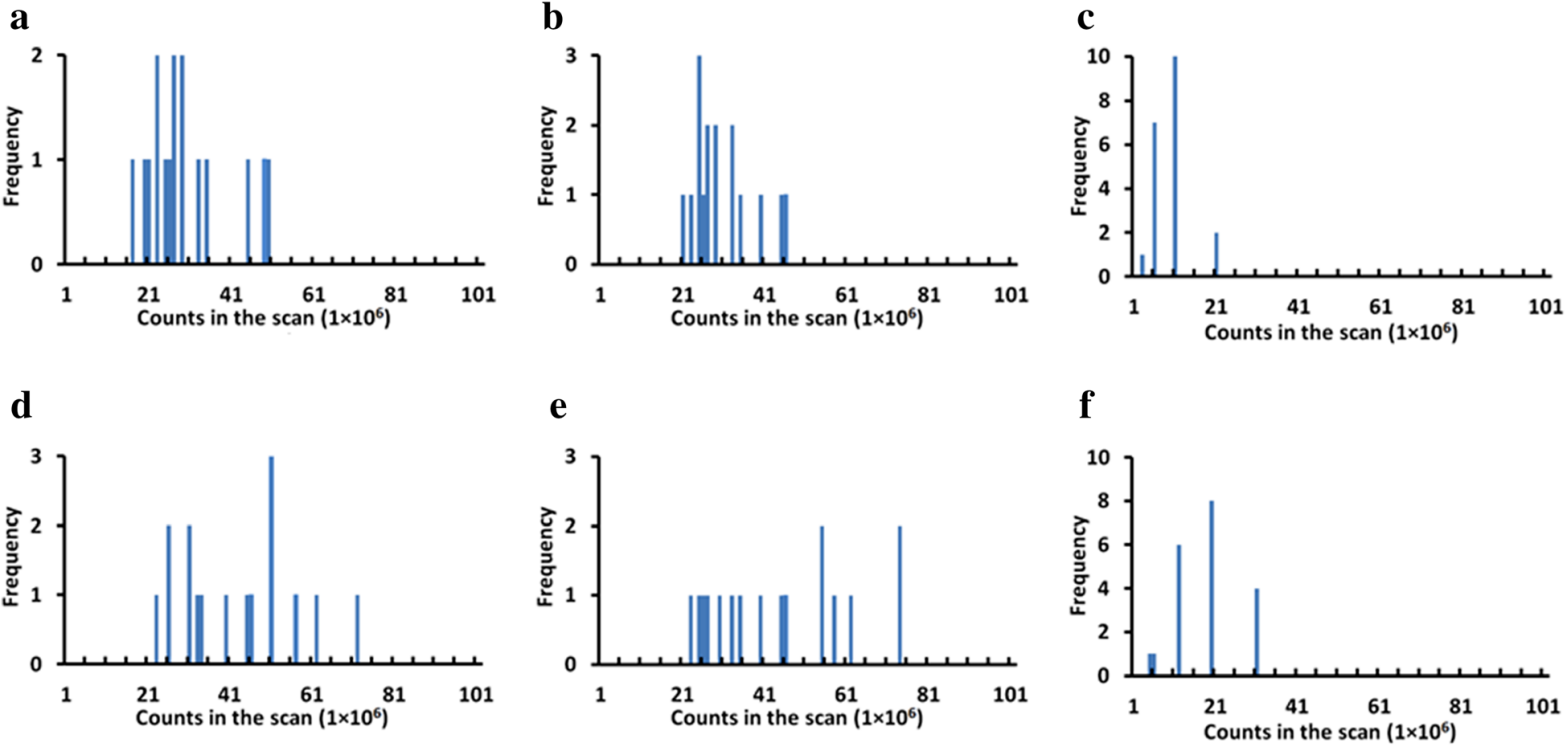
Frequency of cases with COV over 10 % based on SUVmean vs. counts in the scan, in different regions: (**a**) background liver, (**b**) background lung, (**c**) TB lesion and based on SUVmax vs. counts in the scan, in different regions: (**d**) background liver, (**e**) background lung, (**f**) TB lesion. Histogram bin size is 1 × 10^6^

**Fig. 7:**
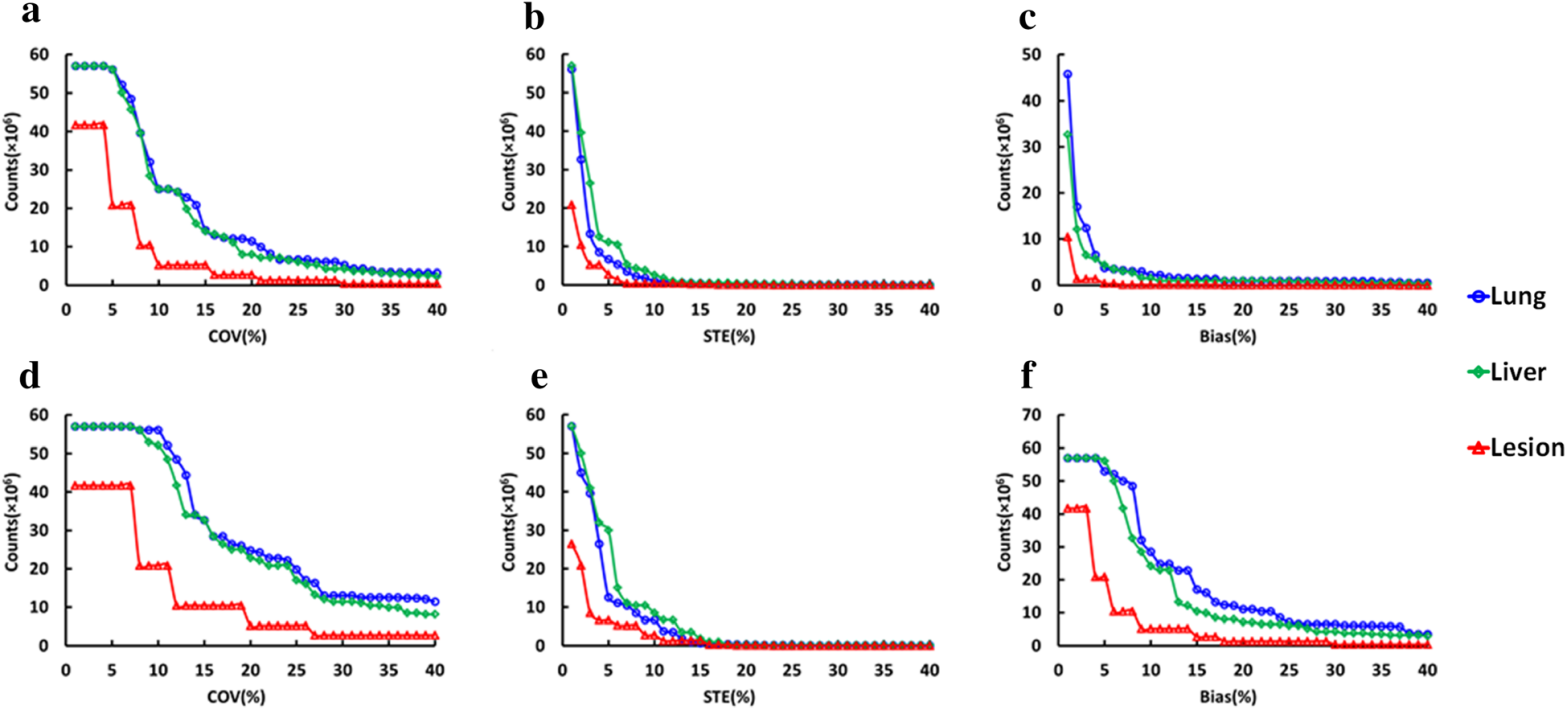
Minimum number of counts needed to keep (**a**) COV for SUVmean, (**b**) STE of SUVmean, (**c**) SUVmean bias, (**d**) COV based on SUVmax, (**e**) STE of SUVmax, and (**f**) SUVmax below a given percent level. VOIs in different regions are shown: background lungs (blue), liver (green), and TB lesions (red)

The variation of the SUV_mean_ and SUVmax over the realizations increases with reducing counts. All patients and lesions were pooled and analyzed in order to study relationship between counts in the scan and two key parameters: measurement bias and error. In Fig. 6, an example of histogram representing the frequency of cases with COV over 10 % at given counts in the scan, in different regions based on SUVmean and SUVmax. Each bin represents the sum of patients (Fig. 6(a, d, b and e)) or lesions (Fig. 6(c and f)) at each count level with COV larger than 10 %. Histograms were created for variable error levels, and the mean and maximum value of the distribution was computed and plotted in Fig. 7(a and d). Similarly, in Fig. 7(b and e), the STE on SUV_mean_ and SUV_max_ is plotted vs. counts level. In Fig. 7(c and f), the measurement bias, defined as difference relative to the full statistics SUV_mean_ and SUV_max_, is plotted vs. counts level.

From Fig. 7(a), the counts required to keep the COV for SUVmean below 10 % for half of the cases in the sample are about 25 × 10^6^, 25 × 10^6^ and 5 × 10^6^, respectively for the liver, lung, and TB lesions. The corresponding counts to keep the COV for SUVmax less than 10 % for half of the cases are about 56 × 10^6^, 52 × 10^6^ and 20 × 10^6^, respectively for the liver, lung, and TB lesions from Fig. 7(d). From Fig. 7(b and e), only 1 × 10^6^, 2.7 × 10^6^ and 0.4 × 10^6^ counts for SUVmean and 6.6 × 10^6^, 8.5 × 10^6^ and 2.8 × 10^6^ counts for SUVmax are needed to reach the STE of 10 % for the liver, normal lung and lesion, respectively. From Fig. 7(c and f), only 2.2 × 10^6^, 1 × 10^6^ and 0.2 × 10^6^ counts for SUVmean and 28.5 × 10^6^, 24.2 × 10^6^ and 5.2 × 10^6^ counts for SUVmax are needed to reach the percentage bias of 10 % for the liver, normal lung and lesion, respectively. At 5 × 10^6^ counts in the scan, bias and noise in terms of SUVmean and SUVmax are up to 10 and 20 %, respectively.

**Fig. 8:**
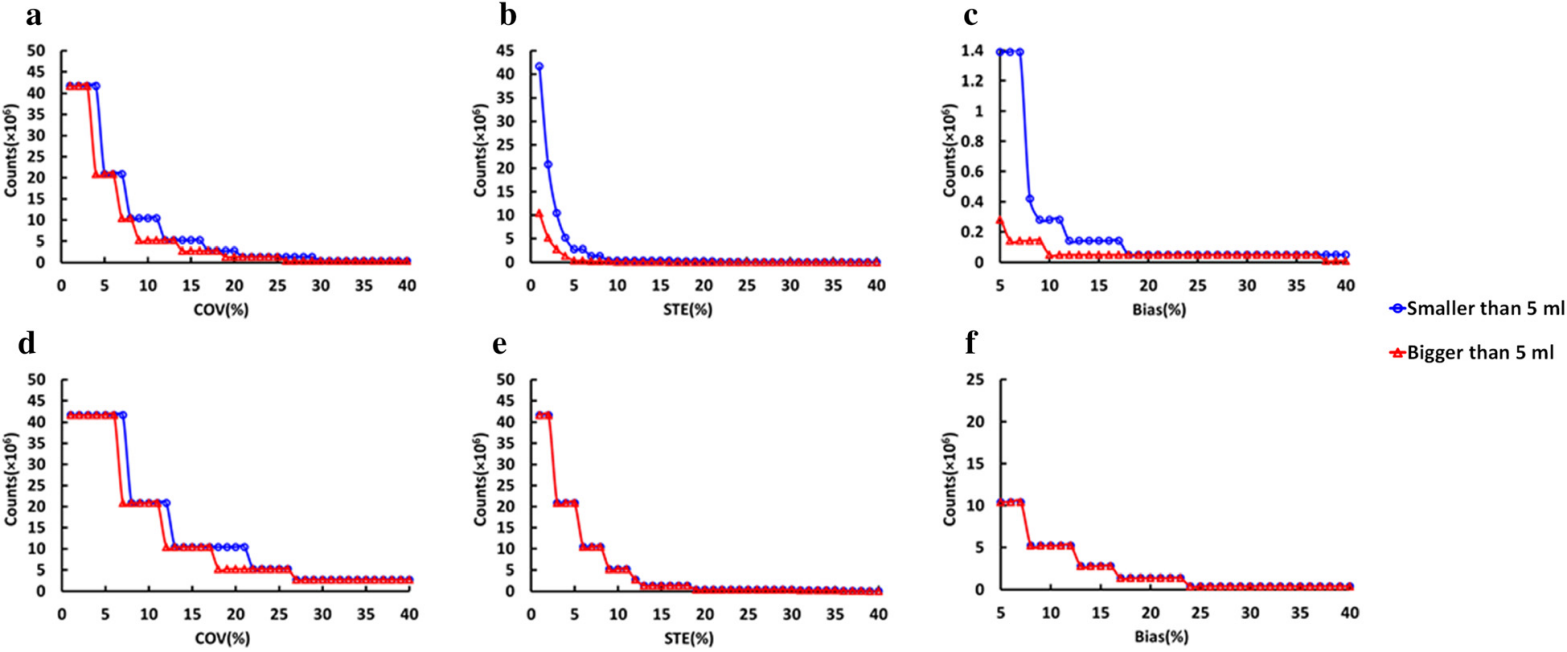
Minimum number of counts needed to keep (**a**) COV for SUVmean, (**b**) STE of SUVmean, (**c**) SUVmean bias, (**d**) COV for SUVmax, (**e**) STE of SUVmax and (**f**) SUVmax bias below a given percent level, for TB lesions. Lesions are separated in two classes: smaller than 5 ml and bigger than 5 ml

The 20 lesions were categorized into two groups based on volume threshold of 5 ml and each group had 10 lesions. One subgroup has mean volume 2.71 ml [1.20–4.81 ml] and another subgroup has mean volume 9.54 ml [5.48–17.58 ml], approximately 17.3 mm [13.2 mm-21 mm] and 26.3 mm [22 mm-32.2 mm] in diameter, respectively. The counts needed to reach the same bias, STE and COV percentage for large lesions (volume ≥ 5 ml) are fewer than that for small lesions (volume ≤ 5 ml) for SUVmean, although the difference between the two groups is small. In addition, the bias and STE for SUVmax were the same for the two groups, as shown in Fig. 8.

## Discussion

We evaluated image quality with objective metrics including SNR in the liver, CNR in the lesions, bias and noise in the liver, normal lung and lesions, at simulated reduced doses, using ^18^F-FDG PET data at various count levels from TB patients. The underlying biology of TB is different from that of lung cancer, but for a technical study such as this, the uptake levels in TB lesions will be more representative of early stage lung cancer lesions than those in more advanced lung cancer. This work will lay the foundation to determine the appropriate dose or scan time for a future prospective study with lung cancer patients PET/CT scanning.

Accurate delineation of lesions is a prerequisite for quantification of FDG uptake. Although a large number of approaches have been proposed to segment tumors in PET images including threshold based, gradient based [21], and fuzzy Bayesian based methods [22], accurate tumor segmentation is still a challenging task. This is due to limited spatial resolution and the relatively high noise level in PET images, and this process evidently becomes more challenging with fewer counts (Zaidi and El Naqa, [23]). A simple thresholding method was employed here to segment the solitary lesions in the lung using the full statistics images and the resulting VOIs were copied to the images at the lower count levels. This simple thresholding method may lead to imperfect delineation of the tumor. In addition, the spill-out from the tumor to the surrounding background can lead to lower CNR. However, the inaccurate delineation will not change the behavior of the image metrics since the error will have the same effect at the different count levels, which is partially supported by the result of CNR varying with different threshold (20, 40, 60 and 80 % of SUVmax). Since we work towards low dose PET imaging for those patients at high risk who have indefinite findings with low dose CT screening, VOIs can be delineated on the CT image and copied onto registered PET images.

Figure 1(a) is the plot of SNR^2^ in the liver for all patients at all count levels and Fig. 1(d) shows the *K*_*i*_ for all patients derived from the SNR^2^ in the liver for each patient at all count level. The SNR in the liver depends on many factors including scanner sensitivity, administered dose, scan time and patient-dependent parameter such as weight. In this study, after adjusting the effect of patient’s weight, the SNR^2^ was found to have a very good linear relationship with detected counts (y = 1.00 × + 0.43, R^2^ = 0.99), which fits with Poisson statistics of nuclear positron emission [18, 19]. At very low counts, the Poisson statistics approximation does not hold anymore, and SNR^2^ acquires a sharper decrease as shown in Fig. 1(b and c), and in Fig. 1(e and f). Since the SNR^2^ in the liver will become nonlinear at very low count levels, the slope of the fit for the count range of 0–1 × 10^6^ will be different from that of the fit at the count range of 0–20 × 10^6^. As expected, also the CNR in the lesion decreases with decreasing counts. After being normalized to CNR at full count data, a function can be used to predict CNR at different count level. A heuristic curve that best fits the CNR was used. In this phase, no interpretative model is proposed for the behavior of the curve, but we applied the simplest function that could fit the experimental data and allow for the prediction of CNR at low counts.

A consequence of dose or count reduction is a possible bias in SUVmean or SUVmax measurement, and a larger error in the measurement, or degradation of measurement reliability, and an increase of noise in the image, which affects detectability of small lesions. This effect has been studied and a bias has been observed, as well as an increase of noise as COV, and an increase of STE of the SUVmean and SUVmax measurement in different regions of the patient. Several factors influence this including a positive bias in the cold background and negative bias in the hot regions associated with the positivity constraint of the OSEM reconstruction for SUVmean. The SUVmax is easily impacted by count reduction than SUVmean. In Fig. 5, one can observe larger error bars and SUV instability at very low counts.

As shown in Fig. 7, fewer counts in the scan correspond to higher COV, STE or error of the radioactivity measurement for both SUVmean and SUVmax, as well as a higher bias relative to high count rate. In comparison with SUVmean, more counts are needed to keep the same acceptable level for COV, bias and STE for SUVmax. In terms of counts required to obtain the same percent error, fewer counts are needed for lesions than for liver and lung background. For example, if the acceptable level for COV for SUVmean is 10 %, the corresponding required counts are 5 × 10^6^, 25 × 10^6^, and 25 × 10^6^, respectively for TB lesions, liver and normal lungs (Fig. 7(a)). This can be explained by the inherent “real” local variations of uptake values in large VOIs in lungs and liver, which are clearly not uniform. In addition, the local variations can explain the different appearance of histograms in the Fig. 6. The number of counts to maintain the same COV for SUVmax are 20 × 10^6^, 56 × 10^6^ and 52 × 10^6^ for TB lesions, liver and normal lungs (Fig. 7(d)). The actual variations add to the statistical noise. Apparently, bias for SUVmean is less sensitive to count statistics, and minimum bias can be reached even below 1 × 10^6^ counts. In addition, the same acceptable level for STE regarding to SUVmean can be maintained with fewer counts as compared to COV, which is demonstrated by 0.4 × 10^6^, 2.7 × 10^6^ and 1 × 10^6^ counts required to reach STE threshold of 10 % for TB lesions, liver and normal lungs, respectively (Fig. 7(b)).

Preliminary investigations show that the behavior of SUVmax is similar, but SUVmax is much more sensitive to noise, and comparable levels can be reached only with much higher number of counts in the scan. An additional study was done by splitting the lesion group into two subgroups: small lesions, with volume smaller than 5 ml; and large lesions, with volumes greater than 5 ml. Each subgroup has 10 lesions. As demonstrated in Fig. 7, for a given number of counts in the scan, bias, COV and STE are larger for smaller volume lesions. Regarding to SUVmean, choosing a target number of counts of about 5 × 10^6^ counts, a noise level (COV) of 9 % is obtained for large lesions, and of 12 % for small lesions. A STE of 4 and 2 % are reached at 5 × 10^6^ counts for small lesions and large lesions, respectively. Finally, the bias at 5 × 10^6^ counts for both large lesions and small lesions are less than 2 %. At the same count level of 5 × 10^6^ counts, lesion CNR is about 60 % of value at the full statistics level (data in Fig. 3(d)) and SNR in the liver is about 3 (data from Fig. 1(b)).

The independent realizations at different low dose were obtained by randomly discarding the events in the list mode data stream, based on the desired count level and thus these realizations are not fully independent. In addition, we got very similar results with bootstrap resampling [24, 25], which was considered as a better method to produce independent realizations. For example, the fitted function for the SNR^2^ in the liver for the images reconstructed with fewer than 1 million true counts *y* = 2.85*x* + 0.19, *R*^2^ = 0.81, which is close to that function (*y* = 2.9*x* + 0.20, *R*^2^ = 0.79) with the current simulation strategy. This could be due to the fact that most of the image metrics in this work were based on the mean value across these realizations. Therefore, in order to keep in line with our previous study [15], the results with the simulations by randomly discarding the events in the list mode data stream were presented in this work.

In the earlier works by Budinger, TF, et al. [26], Hoffman, EJ, et al. [27] and Strother, SC, et al. [28], the relationship between image counts and noise (root-mean-square) had been investigated. These earlier works used a different reconstruction scheme and our findings cannot be compared directly. Notwithstanding this, the statistical noise (COV) in the liver for each subject at the different count level in this study was close to the root-mean square calculated the equation found in the earlier studies up to the count level of 5 million.

In this work, we chose OSEM reconstruction with 3 iterations, 21 subsets and post reconstruction Gaussian smoothing with FWHM of 5 mm which are commonly used parameters in the clinical settings. In future, we will investigate the optimization of the reconstruction parameters with prospective lung cancer patient data once the impact on image quality of reducing counts is generally understood. In addition, the impact of inaccurate attenuation map on quantitative PET lung imaging due to respiratory motion will be thoroughly explored with the data at different counts levels.

## Conclusions

We developed a method and the tools for objectively evaluating PET images at various count levels with ^18^F-FDG PET data of TB patients acquired on a combined PET/MR scanner. This work allowed us to quantify the loss of SNR and CNR as a function of the counts in the scan, in turn related to dose injected. At 5 × 10^6^ counts in the scan, the average SNR in the liver in the observed samples is about 3, and the CNR is reduced to 60 % of value at the full statistics level. At the count level of 5 × 10^6^, bias and noise in terms of SUVmean and SUVmax are up to 10 and 20 %, respectively. This initial investigation presents the first step in a comprehensive image analysis of low dose PET imaging and lays the foundation for future study on low dose PET imaging for lung cancer, which will extend analysis to a larger and lung cancer patient sample, and assess if image quality at reduced counts is sufficient for lung cancer screening.

## Abbreviations

CNR: Contrast-to-noise ratio
COV: Coefficient of variation
OP-OSEM: Ordinary poisson ordered subsets expectation maximization
PSF: Point spread function
SD: Standard deviation
SNR: Signal-to-noise ratio
SUV: Standardized uptake value
SUVmaxm: Maximum SUV
SUVmean: Mean SUV
TB: Tuberculosis
VOI: Volumes of interest
