# Erratum to: A method to assess image quality for Low-dose PET: analysis of SNR, CNR, bias and image noise

**DOI:** 10.1186/s40644-016-0094-0

**Published:** 2016-10-19

**Authors:** Jianhua Yan, Josh Schaefferkoetter, Maurizio Conti, David Townsend

**Affiliations:** 1 Department of Nuclear Medicine, First Hospital of Shanxi Medical University, 85 Jiefang S Rd, Yingze, Taiyuan, Shanxi 030001 China; 2 Molecular Imaging Precision Medicine Collaborative Innovation Center, Shanxi Medical University, 85 Jiefang S Rd, Yingze, Taiyuan, Shanxi 030001 China; 3 A*STAR-NUS, Clinical Imaging Research Center, Center for Translational Medicine, 14 Medical Drive, #B1-01, 17599 Singapore, Singapore; 4 Department of Diagnostic Radiology, National University Hospital, Main Building, 5 Lower Kent Ridge Road, Level 3, 119074 Singapore, Singapore; 5 Siemens Healthcare Molecular Imaging, 810 Innovation Drive, Knoxville, TN37932 USA

## Erratum

After publication of the original article [1], it was noted that one of the author’s names was presented incorrectly. The correct name of the author (Josh Schaefferkoetter) has been updated in the original article, and published in this Erratum for quick reference. We apologise for any confusion this may have caused.
